# Unilateral vs. bilateral DLPFC rTMS: comparative effects on depression, visual-spatial memory, inhibitory control and cognitive flexibility in major depressive disorder

**DOI:** 10.3389/fpsyt.2024.1400414

**Published:** 2024-09-03

**Authors:** Fatemeh Asgharian Asl, Sajjad Abbaszade, Horeyeh Derakhshani, Ladan Vaghef, Amirreza Asgharian Asl

**Affiliations:** ^1^ Department of Cognitive Neuroscience, University of Tabriz, Tabriz, Iran; ^2^ Department of Cognitive Science, Faculty of Education & Psychology, University of Tehran, Tehran, Iran; ^3^ Research Center for Convergent Technologies, University of Tehran, Tehran, Iran; ^4^ Faculty of Psychology and Education, Kharazmi University, Tehran, Iran; ^5^ Department of Psychology, Faculty of Education & Psychology, Azarbaijan Shahid Madani University, Tabriz, Iran; ^6^ Department of Computer Engineering, Faculty of Electrical and Computer Engineering, University of Tabriz, Tabriz, Iran

**Keywords:** major depressive disorder, transcranial magnetic stimulation, dorsolateral prefrontal cortex, cognition, depression, inhibition, memory, cognitive flexibility

## Abstract

**Background:**

Exciting left DLPFC activity with high frequency and inhibiting right DLPFC with low frequency repetitive transcranial magnetic stimulation (rTMS) has shown antidepressant effects in major depressive disorder (MDD) and executive functions. However, few studies have directly compared unilateral and bilateral protocols.

**Methods:**

Forty-seven individuals with treatment-resistant MDD underwent 10 sessions of rTMS over left DLPFC (20 Hz), bilateral DLPFC (left 20 Hz, right 1 Hz), or sham stimulation. Outcomes were depression (Beck Depression Inventory-II), visual-spatial memory (Corsi Block Test), response inhibition (Go/No-Go task), and cognitive flexibility (Wisconsin Card Sorting Test) assessed before and after treatment.

**Results:**

Both unilateral and bilateral rTMS significantly reduced depression levels versus sham controls based on BDI-II scores. While bilateral stimulation did not improve Corsi Test performance, unilateral protocol enhanced visual-spatial memory. On the Go/No-Go task, accuracy was higher in both active stimulation groups compared to sham, with no response time differences. Neither unilateral nor bilateral rTMS had significant effects on cognitive flexibility per the WCST.

**Conclusions:**

Despite comparable antidepressant effects, unilateral stimulation had some cognitive advantages over bilateral rTMS, potentially due to greater left dorsolateral prefrontal cortex excitation. Further research on parameter optimization is warranted.

## 1 Introduction

Major depressive disorder (MDD) is characterized by persistent low mood, loss of interest, and cognitive and physical symptoms (1). Converging evidence has revealed abnormalities in several brain regions linked to emotional and cognitive processing in MDD, including the dorsolateral prefrontal cortex (DLPFC) (2). Specifically, hypo-activity in the left DLPFC is consistently found in MDD and correlates with symptom severity (3, 4). As the DLPFC is critical for executive functions, left DLPFC hypo-activation likely contributes to the cognitive deficits frequently observed in MDD (5).

Emerging evidence also indicates hyperactivity in the right DLPFC in MDD, which has been associated with negative mood and cognitive impairments (6) (6–9). This suggests bi-hemispheric alterations in DLPFC function in MDD, with hypo-frontality in the left DLPFC and hyper-frontality in the right DLPFC. Neuromodulatory techniques like repetitive transcranial magnetic stimulation (rTMS) allow direct modulation of cortical activity in targeted regions. High-frequency (HF) rTMS applied to the left DLPFC has shown antidepressant efficacy, potentially by correcting left hypo-activity (10, 11). Low-frequency rTMS over the right DLPFC has been proposed to potentially reduce hyperactivity in this region and alleviate associated negative cognitive and affective symptoms in major depressive disorder, such as impaired executive functioning, rumination, and persistent negative mood (8, 12, 13). Bilateral protocols incorporating left HF-rTMS and right low-frequency rTMS may have additive benefits in MDD by normalizing the bi-hemispheric imbalance (13). However, most studies have focused on unilateral left stimulation, with limited direct comparisons of bilateral and unilateral rTMS, especially on cognition. Elucidating differences between unilateral and bilateral neuromodulation can provide insights into optimizing protocols (14).

Here, we investigated and compared the effects of unilateral HF-rTMS over left DLPFC versus bilateral rTMS (left high-frequency DLPFC and right low-frequency DLPFC stimulation) on depression severity, visual-spatial memory, response inhibition, and cognitive flexibility in MDD. We hypothesized bilateral rTMS would enhance cognition along with similar antidepressant efficacy as unilateral protocol.

The primary hypothesis was that bilateral repetitive transcranial magnetic stimulation (rTMS), combining high-frequency (HF) stimulation over the left dorsolateral prefrontal cortex (DLPFC) and low-frequency (LF) stimulation over the right DLPFC, would enhance cognitive functioning in major depressive disorder (MDD) patients. Specifically, it was hypothesized that bilateral rTMS would improve visual-spatial memory performance, response inhibition accuracy, and cognitive flexibility. Additionally, it was postulated that bilateral rTMS would demonstrate comparable antidepressant efficacy to unilateral HF-rTMS over the left DLPFC alone.

## 2 Materials and methods

### 2.1 Participants

Sixty right-handed outpatients (43 female and 17 male, age range 18-53 years) meeting DSM-5 criteria for non-psychotic major depressive disorder participated in this study. All participants underwent a structured clinical interview by psychiatrists. Patients had failed to respond to at least two antidepressant medications given in adequate doses. Exclusion criteria included neurological or neurodevelopmental disorders, history of seizures, serious medical conditions, substance use disorders, or previous treatment with TMS. Participants provided written informed consent before enrollment. This study was conducted according to the guidelines of the Declaration of Helsinki and was approved by the Ethics Committee of Tabriz University of Medical Sciences.

### 2.2 Locating the dorsolateral prefrontal cortex

**Figure 1:**
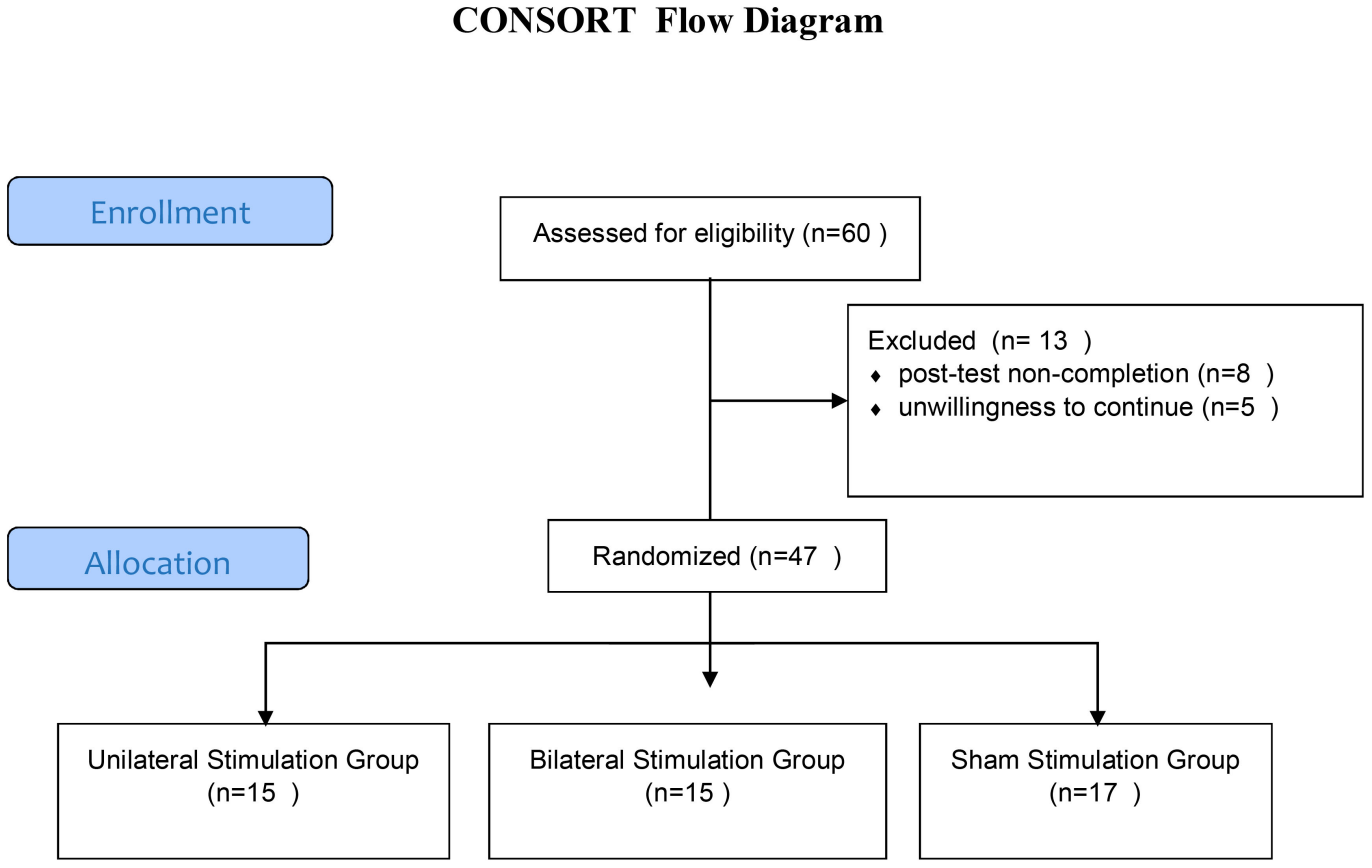
Consolidated Standards of Reporting Trials (CONSORT) flow chart for trial recruitment.

The dorsolateral prefrontal cortex (DLPFC) was localized using the 5-cm rule, one of the most commonly employed methods in rTMS research and clinical practice (49, 15). Specifically, the DLPFC site was determined by moving the TMS coil 5 cm anterior from the motor hotspot for the abductor pollicis brevis muscle of the contralateral hand. This pragmatic method has demonstrated reasonable accuracy in targeting the DLPFC compared to neuronavigational approaches (15, 50). The recruitment and flow of participants through the trial is illustrated in **Figure 1**.

### 2.3 rTMS protocol

Subjects were randomly allocated to three groups (n=20 per group) to receive active unilateral rTMS, active bilateral rTMS, or sham rTMS.

Repetitive TMS was delivered using a figure-eight coil (70 mm diameter) connected to a Magstim Super Rapid magnetic stimulator [Magstim Ltd, UK]. The resting motor threshold (RMT) was determined as the minimum TMS intensity over the left motor cortex needed to evoke a motor-evoked potential ≥50 μV in the right abductor pollicis brevis muscle on at least 5 out of 10 trials (16).

In the unilateral protocol, 2,200 pulses were applied at 20 Hz frequency and 85% RMT intensity over the left dorsolateral prefrontal cortex (DLPFC). In the bilateral protocol, 1,400 pulses were delivered to the left DLPFC at 20 Hz frequency and 85% RMT along with 800 pulses to the right DLPFC at 1 Hz frequency and 110% RMT intensity. For sham stimulation, the coil was angled 90° off the scalp to direct the magnetic field away from the brain, mimicking the auditory and sensory experience of active rTMS without actual cortical stimulation. The TMS course consisted of 10 sessions conducted on weekdays over 2 weeks. Clinical evaluations and cognitive testing were performed at baseline before the first TMS session and again after the final session by examiners blinded to the stimulation protocol.

While most clinical trials investigating rTMS for depression employ 20-30 sessions, our study utilized a 10-session protocol based on evidence suggesting that a shorter course of high-frequency rTMS over the left DLPFC can be effective in alleviating depressive symptoms (51, 52). Furthermore, cognitive effects of rTMS have been observed after as few as 10 sessions in some studies (17, 18). The decision to use a 10-session protocol was also influenced by practical considerations, such as patient burden and resource limitations, common in clinical settings.

### 2.4 Beck depression inventory-II

The levels of depression were assessed before and after rTMS intervention using the Beck Depression Inventory-II (BDI-II). The scoring, categorized from 1 to 6, denoted the range from minimal to maximal depression. Based on the score classifications, scores between 0-10 were considered normal (1), 11-16 indicated mild depression (2), 17-20 suggested the need for consultation with a psychiatrist (3), 21-30 signified moderate depression (4), 31-40 indicated severe depression (5), and scores exceeding 40 represented extremely severe depression (6).

### 2.5 Go/No-Go task

The Go/No-Go Task (GNGT) was administered using the Psychology Experiment Building Language (PEBL) software. The task consisted of two parts, each with an equal number of “go” and “no-go” trials (1:1 ratio), with 100 trials per part (50 “go” trials and 50 “no-go” trials presented in a randomized order). Visual stimuli (letters P and R) were displayed on the screen for 500 milliseconds, with an inter-stimulus interval of 1000 milliseconds. In the first part, participants had to press the spacebar as quickly and accurately as possible when they saw the letter P appear on screen, but not press anything when the letter R appeared. In the second part, they had to press the spacebar for R but not for P. Participants were instructed to respond as quickly and accurately as possible by pressing the spacebar for “go” trials and withholding their response for “no-go” trials. The main measures were accuracy, errors made, and how fast they responded (response time). This tested their ability to respond or inhibit a response depending on the stimulus.

### 2.6 Wisconsin card sorting test

The Wisconsin Card Sorting Test (WCST) computerized version from the PEBL battery was used to evaluate cognitive flexibility (19). For this task, four stimulus cards are presented varying on three dimensions (color, shape, number of items). The participant is then given a stack of response cards and asked to match each one to one of the stimulus cards. After each match, the participant receives feedback indicating whether they matched correctly based on the current sorting rule. However, the sorting rule unpredictably changes after 10 correct matches, requiring the participant to use the feedback to identify the new sorting principle. The test ends after six categories are completed or all 128 cards are used. The main outcome measures are categories completed, perseverative errors (persisting with the old rule), and failure to maintain the set (errors after learning a new rule). Better cognitive flexibility is reflected by more categories completed and fewer perseverative and failure to maintain set errors.

### 2.7 Corsi block-tapping test

Visual-spatial memory was assessed using the Corsi Block-Tapping Test from the PEBL battery. On this task, participants must reproduce sequences of blocks that light up on the screen in varying spans. The outcome was the memory span, reflecting temporary visual-spatial storage capacity. Performance on this task involves actively manipulating visuospatial representations, tapping the hypothesized ‘visual-spatial sketchpad’ component of working memory proposed by Baddeley and Hitch (20). Thus, this test provides a measure of the short-term maintenance of visual and spatial information.

### 2.8 Statistical analysis

The information was processed through both descriptive and inferential statistics. To summarize the study variables, descriptive statistics employed frequency, mean, and standard deviation. In the case of inferential statistics, given the study’s design, analysis of covariance (ANCOVA and MANCOVA) was used to compare the outcome measures between the active and sham groups. Additionally, paired t-tests were carried out to evaluate changes within each group from baseline to post-treatment.

All data were input into SPSS software version 25 for analysis, and the significance level was set at p ≤ 0.05 for hypothesis testing. In cases where statistically significant differences were observed between groups, Bonferroni *post-hoc* tests were conducted. To assess the normality of data distribution, the Kolmogorov–Smirnov test was utilized. Due to unequal variances, a weighted least-squares method was applied for the BDI-II and GNGT scores.

## 3 Results

The original participant sample included 60 individuals who underwent pre-testing. However, 13 subjects dropped out for reasons including post-test non-completion or unwillingness to continue. The final analyzed sample comprised 47 in three groups including unilateral stimulation (n=15; mean age 36.12 ± 9.45 years), bilateral stimulation (n=15; mean age 35.58 ± 10.22 years), and sham stimulation control (n=17; mean age 34.50 ± 9.50 years).

**Figure 2:**
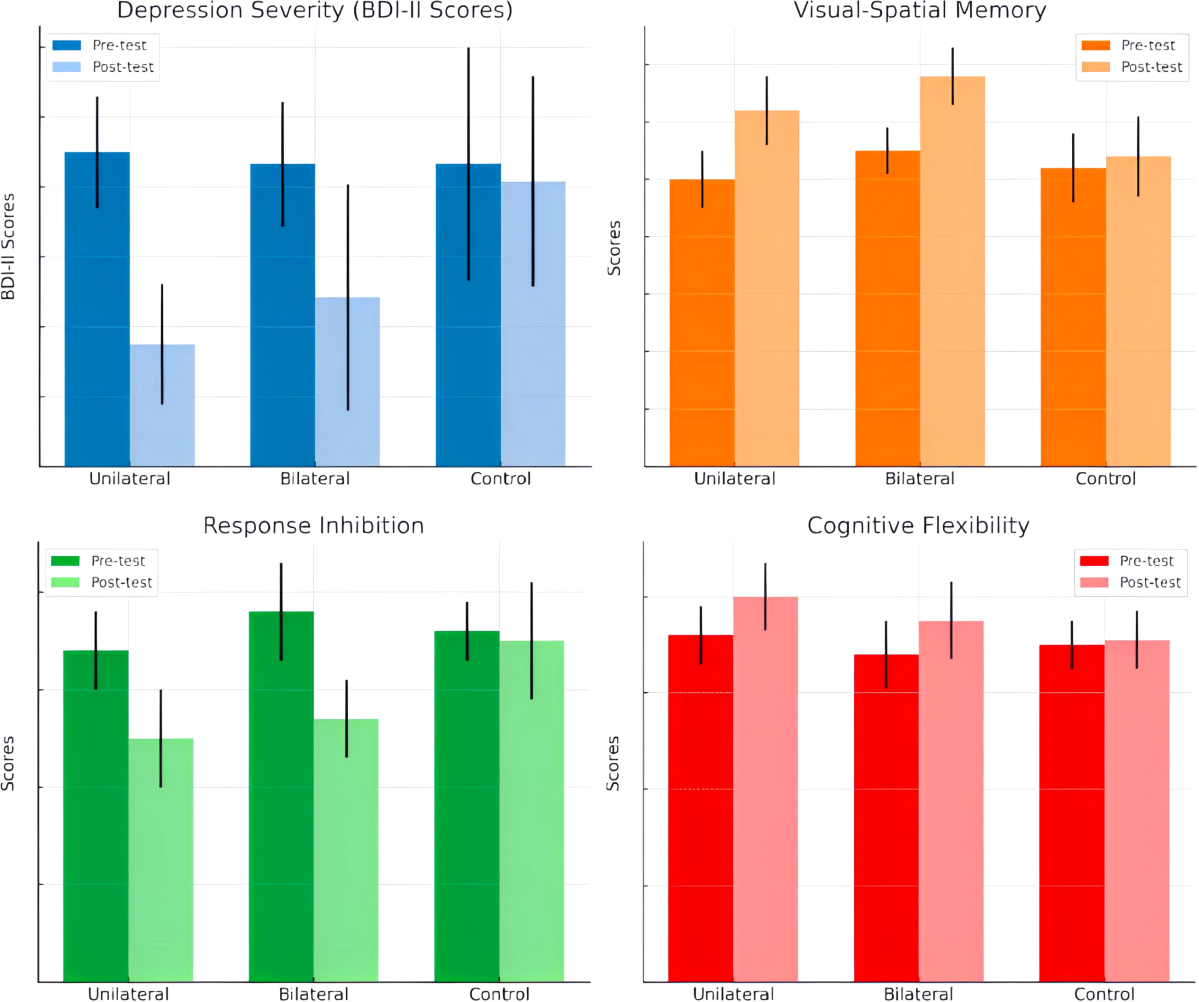
Comparative Effects of Unilateral and Bilateral DLPFC rTMS on Depression Severity, Visual-Spatial Memory, Response Inhibition, and Cognitive Flexibility in Major Depressive Disorder”.

The groups did not significantly differ in age (p >.05). Baseline ANOVA tests found no significant between-group differences in depression severity (BDI-II), visual-spatial memory (CBT), response inhibition (GNGT), or cognitive flexibility (WCST) performance. The comparative effects of unilateral and bilateral DLPFC rTMS on depression severity, visual-spatial memory, response inhibition, and cognitive flexibility in Major Depressive Disorder are presented in **Figure 2**.

### 3.1 Beck depression inventory

The mean BDI-II scores at pre- and post-intervention are displayed in **Table 1**. ANCOVA showed a significant effect of group (F_(2,23)_ =10.241, P<0.05, η2 = .406) (**Table 2**). *Post-hoc* tests indicated significantly decreased BDI-II scores after unilateral and bilateral rTMS compared to sham controls (**Table 3**). Furthermore, paired t-tests revealed significant pre-post differences in BDI-II scores for the unilateral group and the bilateral group but not the sham group (**Table 4**).

**Table 1:** BDI-II scores before and after treatment.

Group	Pre-test		Post-test	
	Mean	SD.	Mean	SD.
Unilateral	34.8	6.3	14.2	7.1
Bilateral	33.9	7.2	20.3	12.8
Sham	33.7	13.2	31.9	11.7

SD, Standard Deviation.

**Table 2:** ANCOVA Results for rTMS effect on depression severity (BDI-II Scores).

Dependent measure		SS	Df	F	Sig.	η2
BDI-II score	Group	1.100	2	10.241	.000	.406
	Error	1.612	30			

**Table 3:** Bonferroni *post-hoc* comparisons of post-treatment depression severity (BDI-II Scores) between groups.

Dependent measure	Comparison	Mean Difference	Sig.
BDI-II score	Bilateral vs. Unilateral	.107	.823
	Sham vs. Unilateral	.419	.000
	Sham vs. Bilateral	.312	.008

The mean difference represents the difference in adjusted post-treatment BDI-II scores between groups.

**Table 4:** Results of paired-sample t-test of rTMS effect on BDI-II scores.

Group	Pre-test		Post-test		t	Sig.
	Mean	SD.	Mean	SD.		
Unilateral	4.5	.798	1.75	0.866	9.869	.000
Bilateral	4.33	.888	2.42	1.621	3.838	.003

### 3.2 Corsi block-tapping test

The mean scores on the Corsi Block-Tapping Test (CBT) are displayed in **Table 5**. ANCOVA results revealed no significant differences between the groups on the CBT (P>0.05) (**Table 6**). However, paired t-tests indicated that while bilateral stimulation did not lead to a significant effect (p>0.05), unilateral stimulation resulted in significant improvement (p<0.05) (**Table 7**).

**Table 5:** Corsi block-tapping test scores before and after treatment.

Group	Pre-test		Post-test	
	Mean	SD.	Mean	SD.
Unilateral	4.000	1.224	4.250	1.453
Bilateral	4.125	1.110	4.583	1.221
Sham	4.000	1.022	3.875	1.025

**Table 6:** ANCOVA Results for rTMS effect on corsi block-tapping test scores.

Dependent measure		SS	Df	F	Sig.	η2
Corsi score	Group	.556	2	1.228	.321	.141
	Error	3.458	15			

**Table 7:** Paired t-test results for rTMS effect on corsi block-tapping test scores before and after treatment.

Group	Pre-test		Post-test		T	Sig.
	Mean	SD.	Mean	SD.		
Unilateral	4.000	1.2247	4.250	1.4538	-2.303	.042
Bilateral	4.125	1.1104	4.583	1.2216	-1.318	.214

### 3.3 Go/No-Go task

The GNGT mean scores are presented in **Table 8**. The MANCOVA results indicated no significant differences between the groups on response time measures in the response inhibition task (p>0.05) (**Table 9**). However, significant differences were observed between the groups for number of correct responses (F_(2,28)_ =7.741, P<0.05, η2 = .356). Follow-up Bonferroni tests were conducted to determine which specific groups differed (**Table 10**).

**Table 8:** Go/No-Go task performance scores before and after treatment.

Variable	Group	Pre-test		Post-test	
		Mean	SD.	Mean	SD.
Total correct	Unilateral	305	11.537	312.83	4.489
	Bilateral	306.67	6.857	312.75	5.119
	Sham	303.33	8.659	303.42	8.533
Response time- Go-round1-P	Unilateral	623.986	120.829	592.573	73.015
	Bilateral	582.651	62.031	606.502	79.619
	Sham	560.003	66.95	544.1328	81.25
Response time- NoGo-round1-R	Unilateral	409.0705	147.943	399.0978	129.02
	Bilateral	478.8708	65.389	472.0623	183.412
	Sham	362.5862	172.881	401.0477	132.692
Response time- NoGo-round2-P	Unilateral	221.7821	236.231	331.0973	273.68
	Bilateral	527.4303	454.023	399.5333	440.017
	Sham	289.6944	259.555	291.345	269.964
Response time- Go-round2-R	Unilateral	645.3955	79.445	621.0592	58.058
	Bilateral	653.5988	52.464	648.9295	61.958
	Sham	629.654	68.168	626.5393	81.881

**Table 9:** MANCOVA results for rTMS Effect on Go/No-Go task performance.

Variable		Df	F	Sig.	η2
Total correct	Group	2	7.741	.002	.356
	Error	28			
Response time- Go-round1-P	Group	2	3.184	.056	.180
	Error	29			
Response time- NoGo-round1-R	Group	2	.380	.6870	.026
	Error	29			
Response time- NoGo-round2-P	Group	2	.466	.632	.031
	Error	29			
Response time- Go-round2-R	Group	2	.532	.5930	.035
	Error	29			

**Table 10:** Bonferroni *post-hoc* comparisons of Go/No-Go task performance between groups.

Variable	Comparison	Mean Difference	Sig.
Total correct	Bilateral vs. Unilateral	.002	1.000
	Sham vs. Unilateral	.011	.003
	Sham vs. Bilateral	.009	.013
Response time- Go-round1-P	Bilateral vs. Unilateral	.037	.148
	Sham vs. Unilateral	.002	1.000
	Sham vs. Bilateral	.039	.110
Response time- NoGo-round1-R	Bilateral vs. Unilateral	.119	1.000
	Sham vs. Unilateral	.115	1.000
	Sham vs. Bilateral	.223	1.000
Response time- NoGo-round2-P	Bilateral vs. Unilateral	.584	1.000
	Sham vs. Unilateral	.527	1.000
	Sham vs. Bilateral	.057	1.000
Response time- Go-round2-R	Bilateral vs. Unilateral	.009	1.000
	Sham vs. Unilateral	.008	1.000
	Sham vs. Bilateral	.017	.942

The findings showed no significant differences in Go/No-Go response times between any of the groups. However, the number of correct responses significantly differed between the control group and the unilateral group, as well as between the control group and the bilateral group. Adjusted mean values for the number of correct responses were significantly higher in both the unilateral and bilateral stimulation groups compared to controls. This suggests that rTMS over the DLPFC enhanced response inhibition accuracy regardless of whether a unilateral or bilateral protocol was utilized. No effects on response speed were found.

As shown in the paired t-test table, the p-value for the number of correct responses is less than 0.05 for both the unilateral and bilateral protocol groups. This indicates that these protocols led to a statistically significant difference in post-test scores compared to pre-test scores on the measure of number of correct responses in these groups (**Table 11**).

**Table 11:** Paired t-test results for rTMS Effect on Go/No-Go task performance.

Variable	Group	T	Sig.
Total correct	Unilateral	-2.603	.025
	Bilateral	-2.723	.020
Response time- Go-round1-P	Unilateral	1.534	.153
	Bilateral	-1.471	.169
Response time- NoGo-round1-R	Unilateral	.465	.651
	Bilateral	.147	.886
Response time- NoGo-round2-P	Unilateral	-.974	.351
	Bilateral	.652	.528
Response time- Go-round2-R	Unilateral	1.258	.234
	Bilateral	.334	.745

### 3.4 Wisconsin card sorting test

WCST performance is shown in **Table 12**. The MANCOVA test results showed that there is no statistically significant difference between the two groups in any of the cognitive flexibility subcomponents (p > 0.05) (**Table 13**). Additionally, paired t-tests showed the p-value obtained for all subscales was greater than 0.05 (**Table 14**). This indicates that neither of the two stimulation protocols led to significant differences in post-test scores compared to pre-test on any of these measures in either group.

**Table 12:** Wisconsin card sorting test (WCST) scores before and after treatment.

Variable	Group	Pre-test		Post-test	
		Mean	SD.	Mean	SD.
Categories Completed/Experienced	Unilateral	.6768	.101	.7596	.055
	Bilateral	.5850	.293	.7336	.113
	Sham	.7167	.097	.7592	.063
Correct Responses	Unilateral	40.33	10.360	45.65	8.026
	Bilateral	39.17	13.065	43.83	11.013
	Sham	41.50	8.296	42.25	5.643
Total Errors	Unilateral	23.67	10.360	18.33	8.026
	Bilateral	24.75	13.065	21.25	11.013
	Sham	22.50	8.296	21.50	5.643
Perseverative Errors	Unilateral	10.17	5.024	9.75	3.545
	Bilateral	8.67	5.466	7.08	3.397
	Sham	14.50	4.812	12.58	6.259
Non-Perseverative Errors	Unilateral	15	13.443	8.58	5.017
	Bilateral	16.50	16.528	13.33	11.484
	Sham	8.50	4.890	8.17	5.024
Conceptual Level Responses	Unilateral	32.50	13.748	39.58	10.983
	Bilateral	29.83	16.629	36.17	16.163
	Sham	32.83	11.456	38.17	13.341

**Table 13:** Results of MANCOVA analysis of rTMS effect on WCST scores.

Variable		df	F	Sig.	η2
Categories Completed/Experienced	Group	2	.159	.854	.012
	Error	27			
Correct Responses	Group	2	.400	.674	.029
	Error	27			
Total Errors	Group	2	.505	.609	.036
	Error	27			
Perseverative Errors	Group	2	1.730	.796	.114
	Error	27			
Non-Perseverative Errors	Group	2	.366	.697	.026
	Error	27			
Conceptual Level Responses	Group	2	.160	.984	.001
	Error	27			

**Table 14:** Results of paired-sample t-test of rTMS effect on WCST scores.

Varible	Group	t	Sig.
Categories Completed/Experienced	Unilateral	-2.064	.063
	Bilateral	-1.847	.092
Correct Responses	Unilateral	-1.967	.075
	Bilateral	-1.375	.197
Total Errors	Unilateral	1.967	.075
	Bilateral	.960	.358
Errors Perseverative	Unilateral	.422	.681
	Bilateral	1.003	.337
Errors Non-Perseverative	Unilateral	1.635	.130
	Bilateral	.878	.399
Conceptual Level Responses	Unilateral	-1.820	.096
	Bilateral	-1.247	.238

## 4 Discussion

In the present study, we compared the effects of left unilateral versus bilateral rTMS over the DLPFC on depression severity, response inhibition, and cognitive flexibility in MDD patients.

As the DLPFC is critical for executive functions, left DLPFC hypoactivation likely contributes to the cognitive deficits frequently observed in this disorder (21).

Neuromodulatory techniques like repetitive transcranial magnetic stimulation (rTMS) allow direct modulation of cortical excitability and hold promise in ameliorating MDD symptoms (22). This method uses a magnetic field to stimulate nerve cells in targeted brain areas. High-frequency (HF) rTMS enhances cortical excitability and metabolic activity in the stimulated regions (23).

Given the role of left DLPFC hypofunction in MDD, HF-rTMS applied to this region has been extensively studied for its antidepressant effects. A large body of evidence now demonstrates that HF-rTMS over the left DLPFC has significant antidepressant efficacy in MDD, likely by correcting the baseline dysfunction of this area (24, 25). Alongside mood improvements, HF-rTMS could also potentially enhance cognition by modulating left DLPFC activity (17).

While most studies have focused on left DLPFC stimulation, emerging insights into the bilateral nature of prefrontal cortex changes in MDD have prompted interest in bilateral modulation (26). A few studies applying bilateral rTMS over left and right DLPFC have shown positive mood effects in treatment-resistant MDD (26, 27).

However, very few studies have directly compared the antidepressant and cognitive impact of bilateral vs unilateral HF-rTMS protocols. Elucidating the differences can help determine the optimal stimulation approach to address the mood and cognitive deficits in MDD. In the present study, we compared the effects of left unilateral versus bilateral rTMS over the DLPFC on depression severity, response inhibition, and cognitive flexibility in MDD patients. We hypothesized bilateral stimulation would improve executive functioning along with similar antidepressant efficacy as unilateral protocol.

Our study found that both unilateral and bilateral rTMS led to significant reductions in depression levels based on BDI-II scores, with no significant differences between the protocols. This indicates comparable antidepressant effects for uni- and bilateral stimulation, consistent with some prior studies (28, 29). The mood improvements likely arise from modulating left DLPFC activity, a key region implicated in MDD pathophysiology (30).

For visual-spatial memory, while bilateral rTMS did not increase Corsi span, unilateral stimulation elicited significant gains from pre- to post-treatment. Although there are other findings regarding the effect of stimulating the right DLPFC on visuospatial functions, this likely arises from the role of the left DLPFC in manipulating visuospatial representations (31, 32). The unilateral protocol directly targeted this region compared to the bilateral approach. Furthermore, additional posterior brain areas like the parietal cortex contribute to visual-spatial memory (33).

For response inhibition, accuracy on the Go/No-Go task improved following both unilateral and bilateral rTMS. The DLPFC is involved in inhibitory control (34), so the increased left DLPFC activation from unilateral stimulation likely improved this executive function. The bilateral protocol applied high-frequency stimulation to the left DLPFC and low-frequency stimulation to the right DLPFC. This approach is based on the hypothesis that such a protocol could potentially normalize hemispheric imbalances in prefrontal cortex activity that may contribute to cognitive dysfunction in depression (35).

In their framework, Schutter & van Honk propose that high-frequency rTMS over the left DLPFC could enhance activity in this hypoactive region, while low-frequency rTMS over the right DLPFC could reduce hyperactivity. They suggest this bi-hemispheric modulation might help restore prefrontal balance and improve symptoms.

Several studies have suggested that while high-frequency rTMS over the left DLPFC can enhance cognitive functioning, low-frequency (1 Hz) rTMS over the right DLPFC may impair certain cognitive domains (17, 36). The rationale behind our bilateral protocol was to potentially normalize prefrontal asymmetry by upregulating the hypoactive left DLPFC and downregulating the hyperactive right DLPFC in MDD. However, inhibiting right DLPFC activity with low-frequency rTMS could have counteracted some of the cognitive benefits provided by high-frequency left DLPFC stimulation.

Indeed, while both unilateral and bilateral rTMS improved response inhibition accuracy on the Go/No-Go task compared to sham, only the unilateral protocol enhanced visuospatial memory performance on the Corsi Block-Tapping Test. This suggests that the addition of right DLPFC inhibition may have negated visuospatial memory improvements in the bilateral group. The lack of significant effects on cognitive flexibility, as measured by the Wisconsin Card Sorting Test, could also be related to the inhibitory right DLPFC stimulation component.

Previous work has implicated the right DLPFC in various cognitive processes, including working memory, attention, and cognitive control (17, 37). Therefore, suppressing activity in this region with low-frequency rTMS may disrupt these functions. Future studies are needed to clarify the specific cognitive consequences of inhibiting the right DLPFC and delineate the optimal parameters for bilateral rTMS protocols in depression. Approaches that modulate the right DLPFC in a more focal or patterned manner may be preferential.

However, neither protocol significantly impacted cognitive flexibility based on the Wisconsin Card Sorting Test performance. While some studies show rTMS-induced flexibility (38), others report no effects (39), likely because multiple regions beyond just DLPFC contribute to cognitive flexibility (40, 41).

## 5 Limitations

The study had a relatively small sample size and also lacked comprehensive demographic and clinical data, which are caveats to the interpretation and generalizability of our findings. A longer treatment duration with follow-up assessments could better elucidate the therapeutic effects. Given the need to consider individual differences in brain function when hypothesizing the effects of the two protocols, and the inability to simply explain depression as a static phenomenon based solely on the function of select brain regions or their causal relationships, we initially sought to assess and analyze neurophysiological components like brain waves and vital signs before and after the interventions. However, due to the unreliability of the data obtained through these methods because of technical issues with the equipment, we were forced to exclude them.

Our study did not directly measure changes in prefrontal cortex activity or hemispheric balance. Therefore, while our bilateral protocol was designed based on this theoretical framework, we cannot conclude that it normalized our participants’ hemispheric imbalances. Future studies combining rTMS with neuroimaging techniques could provide direct evidence of whether bilateral stimulation alters prefrontal cortex activity patterns in this way.

One of the key limitations of this study is the exclusion of participants who did not complete the secondary assessments from the analysis, despite being actual clinical patients. This per-protocol analysis approach could potentially introduce attrition bias and limit the generalizability of the findings. The intention-to-treat (ITT) principle, which analyzes all randomized participants in their assigned treatment groups regardless of adherence or completion, is generally recommended for clinical trials (42).

By not adhering to the ITT principle, our study may have overestimated the treatment effects or underestimated the potential for adverse events or dropout rates associated with the rTMS protocols (43). Future studies should employ ITT analysis, using appropriate methods for handling missing data, such as multiple imputation or mixed-effects models, to provide a more robust and unbiased assessment of the efficacy and tolerability of unilateral versus bilateral rTMS approaches (43).

Another limitation of this study is the lack of comprehensive reporting and analysis of demographic and clinical variables beyond age. Factors such as sex, education level, employment status, marital status, baseline depression severity, duration of current depressive episode, number of previous episodes, treatment resistance (defined as failure of adequate trials of multiple antidepressant medications), concomitant psychotropic medications, and medical comorbidities could all potentially impact the response to rTMS interventions (15).

Ideally, these variables should have been reported, and their potential influence on the primary and secondary outcomes should have been statistically assessed to ensure that the treatment groups were well-balanced at baseline. If significant differences existed, these variables could have been included as covariates in the analyses. The lack of comprehensive demographic and clinical characterization limits the interpretability and generalizability of our findings.

Future studies should carefully record and report these relevant variables and conduct appropriate statistical analyses to account for potential confounding factors. This would not only enhance the internal validity of the study but also facilitate the identification of potential moderators or predictors of treatment response, which could inform the personalization of rTMS protocols (15)Another limitation of this study is the use of the BDI-II as the primary depression outcome measure, rather than clinician-administered scales like the HDRS or MADRS, which are considered gold standards by regulatory agencies.

Side effects such as headaches, dizziness, and spasms in the facial muscles, albeit very mild and transient, existed in both the dropout participants and the other subjects, but did not have a particular impact on the individuals’ motivation to continue or leave the study, especially since the possibility of these transient side effects had been explained to them before the start of the interventions. The subjects who withdrew from the trial, did not respond to telephone follow-ups and no information is available about them, or those who were available did not state these side effects as the reason for discontinuing the interventions.

It is acknowledged that the observed differences between unilateral and bilateral stimulation may have been influenced by low statistical power. The relatively small sample size (n=15 per active treatment group) could have resulted in insufficient power to detect subtle differences between protocols, particularly for cognitive measures. While significant effects were observed in some domains, such as visual-spatial memory improvements in the unilateral group, it is possible that other real differences remained undetected due to limited statistical power.

## 6 Future Directions

Future studies could further explore optimal rTMS protocols and parameters, such as combining rTMS with cognitive training to potentially enhance cognitive benefits (18). Additionally, an integrative, multidimensional approach could provide insights into depression mechanisms based on objective measures across units of analysis, including cognitive/behavioral functions, brain activity, genetics, and neurochemistry [RDoC framework] (44).

The unilateral protocol’s partial superiority over bilateral stimulation on some outcomes may relate to greater left DLPFC excitation and less right DLPFC inhibition from fewer inhibitory pulses. The dose-response relationship between left DLPFC pulses and effects could be examined further. Elucidating the contribution of right DLPFC inhibition in bilateral protocols represents another area for future research. Overall, comparisons of unilateral versus bilateral rTMS can help optimize protocols for managing depression’s neuropsychiatric burden.

While the BDI-II is a valid and reliable measure of depression severity (53), future studies may benefit from including both self-report and clinician-administered scales to provide a more comprehensive assessment of depressive symptoms (54) and to align more closely with regulatory recommendations. This approach would allow for a more robust evaluation of treatment effects, as seen in recent rTMS studies (45).

Another potential limitation of this study lies in the partial complexity of our statistical approach. While we believe our use of ANCOVA and MANCOVA was justified given the nature of our data and research questions, we acknowledge that simpler statistical methods, such as repeated measures ANOVA or analysis of change scores, might have provided more easily interpretable results. The choice between these methods involves trade-offs between statistical power, control of confounds, and ease of interpretation (46–48).

It is suggested that future studies employ larger sample sizes to enhance statistical power and more definitively compare the efficacy of unilateral versus bilateral rTMS protocols. Such an approach would allow for more robust detection of potential differences in both clinical and cognitive outcomes.

## Data Availability

The raw data supporting the conclusions of this article will be made available by the authors, by request and without undue reservation.
